# Relevance of pre-stimulus oscillatory activity for the perceived valence of emotional facial expressions

**DOI:** 10.1038/s41598-024-69433-0

**Published:** 2024-08-20

**Authors:** Carina Jaap, Michael Rose

**Affiliations:** Department for Systems Neuroscience, NeuroImage Nord, University Medical Center Hamburg Eppendorf, Martinistrasse 52, 20246 Hamburg, Germany

**Keywords:** Emotional facial expression, Pre-stimulus oscillatory activity, Multivariate analysis, Negativity bias, Human behaviour, Neuroscience

## Abstract

The interpretation of emotional facial expressions is crucial in everyday social interactions, and rapid processing of these expressions is necessary. Although extensive research has shed light on the mechanisms involved in facial expression processing, there is limited research on the potential role of the state of neural activity that directly precedes the occurrence of a face. Here, we investigated the potential modulatory role of pre-stimulus oscillatory activity in emotional facial expression processing. We tested emotional facial processing in two experiments, one utilizing artificial and the other natural facial expressions. The participants had to evaluate the emotional valence of the presented ambiguous facial expressions. In a univariate analysis, differences in the oscillation activity of the later rated valence of the faces were observed in both experiments, and these differences were observed even before the presentation of the facial expressions. Importantly, two different multivariate approaches directly supported the relevance of pre-stimulus oscillatory activity by exclusively using pre-stimulus oscillatory data to predict the perceived valence of the latter rated facial expression across the two experiments within as well as across subjects. The behavioral data shows the often observed negativity bias, i.e. ambiguous faces resulted in the tendency to rate them as negative. This negativity bias was related to neural activity modulations in the pre-stimulus period and also within post-stimulus processing related activity. These findings underscore the significance of pre-stimulus oscillatory activity in facial expression processing, indicating a functional role of ongoing neural states that affects the processing of facial expressions and constitute a basis for the well described negativity bias.

## Introduction

Human faces are one of the main visual stimuli we encounter on a daily basis. Alongside information about a person’s identity, facial expressions play a crucial role in social interactions as they provide insight about a person’s affective state^1–3^. As facial expressions contain such valid information and are essential for social communication, humans become very precise in classifying them^3,4^. Not only are humans precise in recognizing emotional facial expressions, they are also fast in processing them^3,5,6^. Previous studies using EEG have shown that effects of emotional facial expressions can be observed as early as 50–100 ms after stimulus presentation^5^. These early effects were found primarily extending until 200 ms posterior to an emotional facial expression^5–13^, and were found for both positive and negative emotional expressions^9,12,14^. Studies using neural imaging have proposed a theory of a close relationship between the amygdala and early facial emotion processing, especially in fear^15,16^. It has been hypothesized that the fast and automatic processing of emotional facial expressions occurs via processing pathways of the amygdala and thalamus^16,17^, amongst other regions. In addition to these early effects, late effects have been observed in emotional face processing, typically emerging around 300–400 ms after stimulus presentation^11,18–20^. It is hypothesized that early processing operates through automatic, bottom-up mechanisms driven by visual processing, whereas later processes are likely influenced by top-down mechanisms^11^.

However, emotional processing is strongly affected by different stimulus- independent factors like mood^21^, and mood dependent disorders like anxiety^22–24^, depression^25^, social connectedness ^26^ and stress^27^. The modulatory effect of stimulus-independent factors on the processing of emotional stimuli can be assumed to be reflected in pre-stimulus neural activity, but this pre-stimulus neural activity has not been directly examined for emotional face processing yet. Nevertheless, evidence from studies manipulating the anticipation of certain subsequent emotional^28,29^ or facial^30^ stimuli suggest that pre-stimulus oscillations differ depending on the anticipated emotional stimulus. Research in other domains has already emphasized the significance of pre-stimulus activity in the processing of forthcoming stimuli. Studies on pain perception, for instance, have revealed that an individual’s brain oscillations preceding the experience of a painful stimulus can notably predict their perception of pain intensity^31,32^. Studies addressing memory encoding^33–36^ or visual perception^33,37^ have also found a modulation of stimulus processing by oscillations preceding the stimulus.

Another important factor to consider in emotion processing is the interpretation bias. While humans demonstrate proficiency in categorizing distinct facial expressions, an interpretation bias may arise under certain conditions. An interpretation bias entails perceiving the presented emotional facial expression as either more positive or more negative than intended^26,38^. To study interpretation bias in face processing, facial stimuli such as morphs between two unambiguous emotions^39^ or faces expressing surprise^23,38^ are often used. These facial expressions entail a unique challenge since they can evoke both positive and negative interpretations. When presenting an ambiguous expression, the ideal interpretation of the rating of these would be evenly split between positive and negative valence. However, ambiguous expressions can elicit an interpretation bias, particularly when no context is given. This bias tends to be negative and is therefore also referred to as negativity bias^38,40^. A negativity bias has been observed in both clinical^25^ as well as healthy populations^26,40,41^.

In clinical populations, i.e. individuals with a mood disorder, an attention bias for negative expression as well as a specific sensitivity for sadness was observed when categorizing facial expressions^25,42,43^. However, with interventions such as a cognitive bias modification, the negativity bias can be modulated as some studies show^44,45^. The ability to modify a negativity bias in mood disorders, such as depression, is crucial since a negative information processing bias is believed to play a major role in the cause and treatment of these disorders^46^. Identifying and adjusting the origins of negativity bias would be a significant advancement in conventional therapeutic approaches. This is particularly important given the unwanted side effects associated with conventional therapeutic substances and their high placebo response^47,48^.

In healthy participants, a negativity bias is observed in studies in which ambiguous stimuli are presented without a specific context^41^. This negativity bias can be modified, e.g. by practicing mindfulness, which leads to more shifts towards positive interpretations of ambiguity^27^. In addition to mindfulness training, age also appears to have a positive effect on the processing of ambiguous emotions in healthy participants^49–51^. Further, it was shown that the manipulation of expectancy prior to an emotional face can also shift the consecutive emotional processing^52^. These results indicate the importance of stimulus-independent processes, and it can be assumed that a relevant aspect of the processes underlying a negativity bias is reflected in pre-stimulus activity. Therefore, we aimed to examine the modulatory impact of pre-stimulus activity on the processing of subsequent emotional facial expressions.

Using EEG, we address the questions of whether pre-stimulus oscillations are relevant for the perceived valence of subsequent ambiguous facial expressions and whether these pre-stimulus oscillations correlate with the commonly found negativity bias. To test this, all participants were presented with facial stimuli that showed surprised facial expressions and were asked to classify the perceived valence of the faces.

We selected two distinct sets of facial stimuli to replicate the study within participants. Consequently, our study consisted of two experiments, with each participant engaging in both. In Experiment 1, we presented artificial faces and in Experiment 2, we present natural human faces from the Karolinska database^53^ (see “Materials and methods” section for more information on the stimuli). Given that both natural and artificial emotional facial expressions are recognized equally well, as observed in a study in which participants rated different emotions in facial expressions displayed on natural as well as artificial faces^54^, we did not anticipate substantial differences between the results of the two experiments. The rationale behind employing two distinct sets of stimuli was to gain a more comprehensive understanding of the potential involvement of pre-stimulus oscillations on subsequent emotion processing across different stimulus attributes. Consequently, the objective was not to investigate the differences between the two experiments but rather to assess the probable involvement of pre-stimulus oscillations on emotion processing independent of the nature of the emotional expression that is subsequently presented.

Therefore, to achieve a more general conclusion about the relevance of pre-stimulus neural activity in latter emotion processing, we used two multivariate classification approaches. One approach was performed at subject- and the other at group-level. The latter was performed to gain insight on a probable participant and stimulus overarching pattern in the pre-stimulus period between differently perceived valences of subsequent presented emotional facial expressions. In both approaches the classifier was trained on pre-stimulus oscillatory activity data of Experiment 1 and tested on pre-stimulus oscillatory activity data of Experiment 2. The multivariate approach also reflects the multiple contributors that can be assumed to constitute emotional processes and corresponding modulatory factors like stress^27^, anxiety^23^ or mood^21^. Therefore, a multivariate approach may provide valuable insights into the nuanced differences of complex emotional processing compared to a univariate approach.

## Materials and methods

### Participants

Fifty-eight participants took part in this study (37 females, age = 26.6 ± 4.1 years). All our methods were carried out in accordance with the relevant ethical guidelines and regulations and the experimental protocols were approved by the Ethics Committee of the General Medical Council Hamburg (PV7022). The participants were recruited via an online job platform. All participants gave their written informed consent prior to the experiment and were paid an expense allowance of 10 €/h. The inclusion criteria for this study were 18–35 years of age, physically and mentally healthy, normal or corrected to normal vision and normal hearing. Further exclusion criteria contained frequent use of medication except for birth control.

In Experiment 1, 11 participants had to be excluded from the final sample (5 due to a lack of remaining trials after pre-processing, 4 due to technical issues and 1 due to excessive movement), resulting a final sample size of 48 participants (33 females, age = 26.6 ± 4.1 years). In Experiment 2, 19 participants had to be excluded from the final sample (7 due to technical issues, 10 due to lack of remaining trials after preprocessing, and 2 due to excessive movement). This resulted in a final sample size of 39 participants (24 females, age = 26.7 ± 4.2 years).

### Procedure

**Figure 1:**
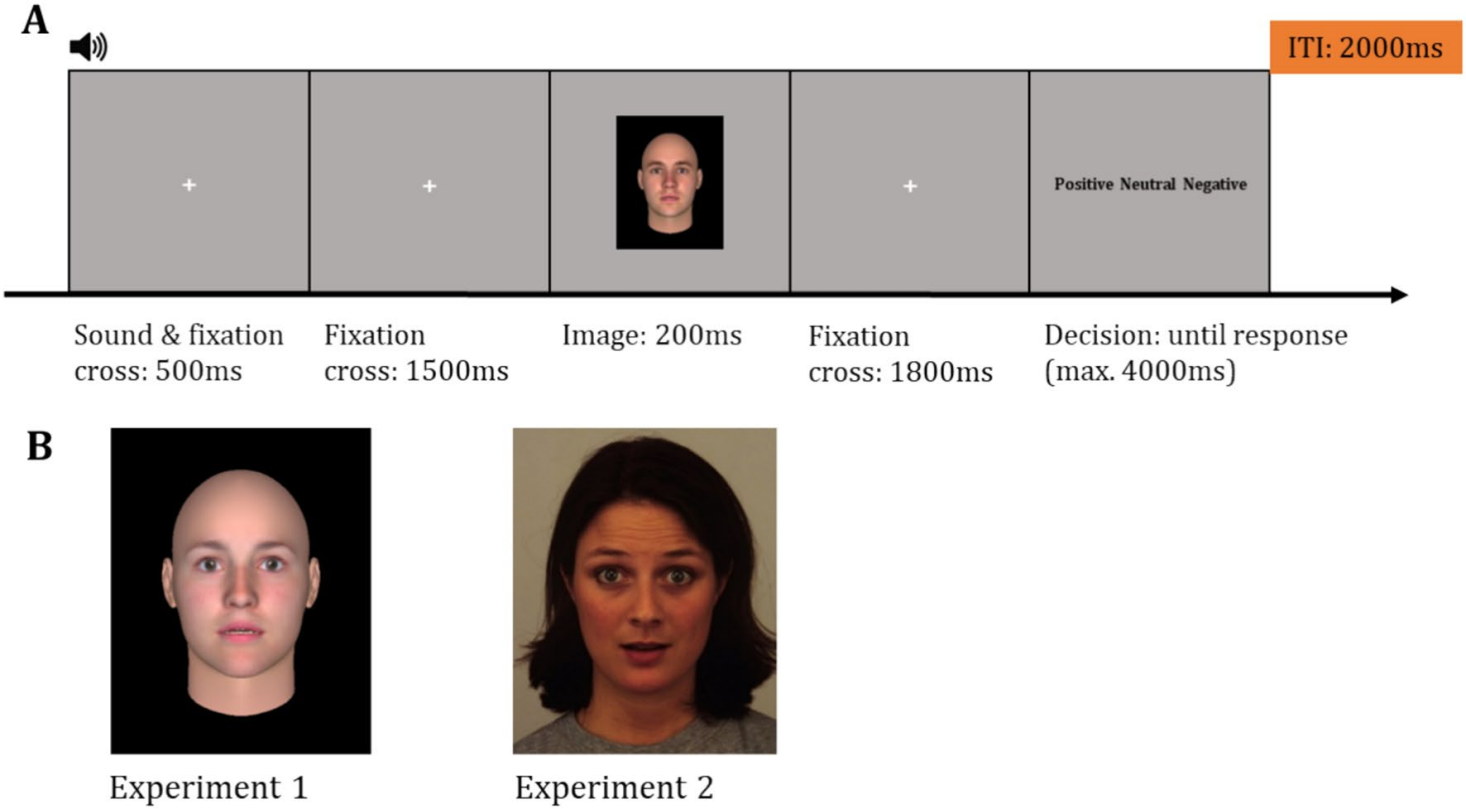
(**A**) Timeline of a trial in the experiment with an example of an image used in Experiment 1. The timeline of trials was identical in both experiments. (**B**) Example images used in the experiments. Experiment 1 contained artificial images of faces created with FaceGen (left) and Experiment 2 natural images of faces from the Karolinska data base^57^ (https://kdef.se/; right, shown here as an example is AF01SUS). All images resemble surprised faces.

After giving written informed consent, each participant filled out the germane versions of the Becks depression inventory 2 with 21 items^55^ (BDI-2) as well as the state trait anxiety inventory with 20 items each^56^ (STAI-Y). After filling out the inventories, the participants were seated onto a comfortable chair in a dimly lit, and shielded room. An oral instruction of the task was given by the experimenter, followed by a written instruction on the computer screen. The participants were instructed to decide whether the perceived valence of a presented face was negative or positive. The participants were also informed about a third choice labelled as ‘neutral’. However, they were also informed that the facial expressions would never be neutral and that they should ideally choose between a positive or negative valence for the facial expression. If the participants were unsure, they were advised to choose ‘neutral’ instead of guessing one of the two valence options. To log their answer, the participants had to click on one of three buttons on a mouse. The participants were instructed to choose between a negative or positive valence using the left and right buttons respectively, while the middle mouse button could be used to choose the neutral option. The buttons for negative and positive valences were marked in red and green respectively. The participants were informed that the task was not a speeded decision task, but that they were expected to make a decision within a maximum of 4000 ms. Each trial began with a 2000 ms presentation of a fixation cross. Then, a neutral sound was played for 500 ms. The fixation cross was still present while the sound played and remained on the screen for 1500 ms after the sound had ended. Next, an image of a face was presented for 200 ms (see Fig. 1). Subsequently, a fixation cross was presented for 1800 ms, followed by the response selection. The participants were instructed to select the valence they perceived on the face. The trial ended when the participant clicked a button on the mouse, or after 4000 ms if there was no response. There was an inter-trial interval (ITI) of 2000 ms between trials. The experiment consisted of two parts. In Experiment 1, 14 different artificial faces were presented in 210 trials (for more information see “Stimuli” section). In Experiment 2, 68 different natural human faces were presented. Each face was only presented once, therefore Experiment 2 consisted of 68 trials. We chose not to repeat the natural faces to avoid possible memory effects of the previously rated valence, since the facial features were quite distinct between the natural faces. The order of the two parts remained the same for each participant, as the real human faces were more salient and might influence how the artificial faces would be perceived. There was a short break between the two experiments and breaks were also provided within each experiment.

### Stimuli

The visual stimuli in Experiment 1 consisted of 14 artificial faces (7 male, 7 female) created with FaceGen (FaceGen Modeller Core, Version 3.22). The age parameter for all generated faces was set to 20, and the nationality was specified as ‘typical European’. To induce a slightly more natural, i.e. imperfect, appearance, an asymmetry value of 0.5 was applied. To create emotional expressions, we used FaceGen’s pre-defined discrete emotional expressions, which have intensities ranging from 0 to 100%. Each of our faces was set to 60% surprise in the FaceGen Modeller. This percentage resulted in a balanced distribution of valence ratings in the pilot measurements. The visual stimuli in Experiment 2 consisted of 68 natural faces (34 male, 34 female) from the Karolinska database^57^. All images consisted of frontal faces from the Karolinska Directed Emotional Faces A series showing surprised faces. The images (6° 17′ 0.77′′) were presented on a computer screen (23″, refresh rate 60 Hz, ~ 1 m distance from the participant). The images and sounds were presented with Presentation® software (Version 22.01, Neurobehavioral Systems, Inc., Berkeley, CA)_._ The neutral sound was created with Audacity® software (Version 2.4.1.) and consisted of a 500 Hz sine wave as a base, with a fade out towards the end of the 500 ms duration. The sound was presented through two speakers (HD 201, Sennheiser, Germany) one on each side of the screen. The volume of the sound was adjusted to a comfortable level for each participant.

### Analysis of behavioral data

The analysis was performed using R (Version 4.3.1). We computed the percentage of negative ratings in relation to all ratings for each participant individually in each experiment in the final sample. Subsequently, we calculated the means and standard deviations of the percentage of negative ratings relative to all ratings separately for each experiment. For the participants in the final sample whose data sets from both experiments were usable for EEG analysis (N = 36), we performed group-level statistics. We performed a paired t-test on the percentage of negative ratings relative to all ratings across both experiments. We then computed a Pearson correlation coefficient between the percentage of negative ratings (in relation to all ratings) and the BDI-2 as well as the STAI within each experiment.

### Analysis of EEG data

#### Acquisition

The EEG signal was recorded from 64 active electrodes (actiCap slim, Brain Products, Gilching, Germany) placed according to the 10–20 system. The recording took place in an electrically shielded and sound-attenuated room. The ground electrode was placed at the inion (Iz). The online reference was at FCz and was later offline re-referenced to a common average reference. To monitor eye movements, three EOGs were placed on the face. Two were placed on the canthi of the eyes and one was placed under the left eye. Fp1 was used as an additional EOG above the left eye. The electrodes were filled with high-viscosity electrolyte-gel for active electrodes (SuperVisc, EasyCap GmbH). Electrode impedances were kept below 10 kΩ. The EEG signal was amplified with a low cutoff frequency of 0.5 Hz and was recorded with a sampling rate of 500 Hz.

#### Preprocessing

The EEG signal was processed offline using the FieldTrip toolbox^58^ (Version 20220632; https://www.fieldtriptoolbox.org/) implemented in MATLAB (Version 2020a, The MathWorks Inc, Natick, Massachusetts, USA). Epochs from − 2000 to 2000 ms relative to the onset of the face stimulus were extracted over the whole dataset. Manual artifact rejection was applied. During manual artifact rejection, we eliminated trials that contained muscle noise, eye blinks in close proximity to the stimulus onset and trials in which the behavioral responses were given too early, i.e. responses given more than 200 ms before the decision window began, which was presented 1800 ms after the presentation of the face ended. Afterwards, an independent component analysis was performed to remove eye movement, blinks, cardiac, and muscle artifacts based on visual inspection of the time course, spectrum, and topography of each component. The re-referencing was then performed followed by another manual artifact detection and rejection followed to remove any remaining artifacts. In rare cases, where a channel was interpolated, the channel was removed prior to the independent component analysis and interpolated after the re-referencing^59^. After the pre-processing was finished, the remaining trials were separated based on the valence response of the participant, i.e. negative and positive ratings of the faces. In Experiment 1, only trials of ambiguous-rated faces, i.e. faces rated as both negative and positive, were considered in the further analysis. In Experiment 1, in which we presented 210 trials in total, a mean of 81 (*SD* = 21) trials remained for the negative and a mean of 68 (*SD* = 28) remained for the positive-rated faces. For the analysis of the time–frequency EEG data, only trial numbers that were equalized between the two perceived valences were used. The trial number of the rated valence with more trials was adjusted to match the trial number of the rated valence with fewer trials by randomly selecting an appropriate number of trials from the larger set of trials (mean number of trials for both valences = 59; *SD* = 20). The inclusion criterion for the analysis of the effective number of trials per rated valence for each participant was 10. In Experiment 2 all natural facial stimuli were presented only once and therefore all remaining trials after the pre-processing were taken into account for the further analysis. In Experiment 2, in which we presented 68 trials in total, a mean of 38 (*SD* = 9) trials remained for the negative and a mean of 19 (*SD* = 6) remained for the positive-rated faces. For the analysis of the time–frequency EEG data, only trial numbers that were equalized between the two perceived valences were used. The trial number of the rated valence with more trials was adjusted to match the trial number of the rated valence with fewer trials by randomly selecting an appropriate subset from the larger set of trials (TFA; mean number of trials in both valences = 19; *SD* = 6). The inclusion criterion for the analysis for the effective number of trials per valence for each participant was 10. We applied the following steps of analysis concerning the time–frequency decomposition consistently across both experiments.

#### Time–frequency decomposition

We conducted a time–frequency decomposition using the method *mtmconvol* in the MATLAB toolbox FieldTrip^58^ (https://www.fieldtriptoolbox.org). The decomposition encompassed frequencies ranging from 2 to 120 Hz with a frequency resolution of 1 Hz for the time-interval of -2000 to 2000 ms. We applied a wavelet convolution with a Hanning taper. A 500 ms sliding window was applied, moving in 50 ms time steps. The temporally and spectrally decomposed data underwent normalization through the application of a baseline correction expressed in decibels (dB). The raw power values were corrected by calculating the ratio between the power values at each time point for each frequency band and the mean power of the baseline period for the same frequency band. The resulting Ratio values were then converted to dB by taking their base 10 logarithm and multiplying by 10^59^. The baseline period used for this approach spanned from − 1500 to − 500 ms relative to stimulus onset. Baseline correction was performed utilizing the implemented function within FieldTrip^58^ toolbox (https://www.fieldtriptoolbox.org).

### Statistical analysis of EEG data

#### Group-level analysis of time–frequency decomposed data

Our aim was to test whether pre-stimulus oscillations are related to processing of later presented ambiguous facial expressions by studying differences of the time–frequency spectra in dependence to perceived valence of emotional facial expressions. For this purpose, two experiments with different stimulus material were performed. The statistical analysis of the time–frequency data was performed separately for each experiment. For the group-level analysis, paired samples t-tests were performed on the power spectrum of the temporally and spectrally decomposed data for the two emotional valences, i.e. negative and positive, using the implemented Fieldtrip function. The calculated t-values were then corrected using nonparametric cluster-based permutation tests as implemented in the Fieldtrip toolbox^60^ (method = montecarlo, cluster threshold = nonparametric-individual, alpha = 0.025, number of randomizations: 2000; frequency: 2 to 60 Hz). We performed the analysis over the time-window of − 500 to 1500 ms relative to stimulus onset.

#### Correlation between oscillatory data and interpretation bias

We aimed to determine whether negativity bias correlates with the differences of the time–frequency spectra of the two perceived valences of facial expressions, independent of the different facial stimuli used in the experiments. We were interested in this correlation irrespective of the different facial stimuli used in the experiments. Therefore, we combined the time–frequency spectra of both experiments. To test for a relation of the negativity bias, i.e. negative ratings in percent in relation to all ratings, and the difference in oscillatory power in perceived valence (negative–positive rated faces), we used the implemented correlation function in FieldTrip^58^ (https://www.fieldtriptoolbox.org). To prepare for the correlation analysis, the difference in oscillatory power between negative and positive rated faces was calculated for each participant for each channel-time–frequency data point in the range of 2–60 Hz and − 500 to 1500 ms relative to stimulus onset. The trials were the same as those used in the main group level analysis, i.e. the number of positive and negative rated trials were equalized within participant. The difference values were then correlated with the percentage of negative ratings (in relation to all ratings) of each participant by computing a Pearson correlation coefficient. The cluster-based permutation correction described in *Group-level analysis of time–frequency decomposed data* was applied to the data to correct for multiple comparisons.

#### Multivariate pattern analysis (MVPA)

In order to test the differences in the time–frequency spectrum in the pre-stimulus time-window in dependence of the perceived valences across both experiments^61^ at subject-level, cross-decoding was performed utilizing the MVPA Light toolbox in Matlab ^62^. The trials from each participant, used in the univariate analysis of each experiment, were also employed for the MVPA. This means, that the trial numbers of each of the two perceived valences (positive and negative) were equalized within participants, as in the univariate group-level analysis. However, no baseline correction was applied to the data, since single-trial data are used for the MVPA and all critical information should remain in the data. Only participants for whom we had data from both experiments were included in the cross decoding analysis (N = 36; mean trials Experiment 1 = 62, *SD* = 20; mean trials Experiment 2 = 19, *SD* = 6).

The data from each participant in Experiment 1 were utilized to train a Support Vector Machine (SVM). The trained SVM was then tested on the data of Experiment 2 from the same participant. The time-window of interest ranged from − 1000 to − 200 ms before visual stimulus onset to ensure that the decoding is based on pure pre-stimulus data. This time-window ensured the testing of purely pre-stimulus-driven differences in perceived valence and allowed for the assessment of the stability of the effect over a large window of 800 ms. In contrast to testing the MVPA over the window of − 2000 to − 1000 ms, this window was chosen to test the activity directly preceding and therefore in direct relation to the onset of the stimulus, without any overlap with the post-stimulus window. On the subject level, the high dimensional time–frequency decomposed data were reduced to two dimensions by averaging across the time domain (channels = 60; frequency = 2–60 Hz). Both channel and frequency served as features for the SVM and accuracy was used as metric**.** The hyperparameter *C* was obtained through grid search implemented in the MVPA Light toolbox.

For the statistical analysis, we performed a binomial test on the accuracy results of each participant using the MVPA Light toolbox^62^. Within the binomial test, a binomial distribution is used to calculate the p-value of each accuracy^61^. The output is a mask, in which all accuracies that reach significance are used for further analyses. Subsequently, we identified the highest accuracy value within this mask for each participant and computed the general mean accuracy of these peak accuracies across all participants. If no values remained within the binomial mask, we utilized the maximum accuracy from the original accuracy results. To test whether the accuracies over all participants are different from chance level, the peak accuracies from the binomial analysis were compared to the chance level of 0.5, since accuracies range between 0 and 1, using a one-sample t-test.

In order to test the differences in the time–frequency spectrum in the pre-stimulus time-window in dependence of the perceived valences across both experiments^61^ on group level, we performed cross-decoding implementing leave-one-participant-outcross-validation (LOPOCV) using the MVPA Light toolbox in Matlab^62^ as a second MVPA approach.

The number of trials, participants, and the pre-stimulus window used for the MVPA at group-level remained the same as for the MVPA performed at subject-level. On the subject level, the high dimensional time–frequency decomposed data were reduced to two dimensions by averaging across the time domain (channels = 60; frequency = 2–60 Hz). The data was then z-transformed. Both channel and frequency served as features for the SVM and accuracy was used as metric. The hyperparameter *C* was obtained through grid search implemented in the MVPA Light toolbox. The SVM was trained with the pooled data of 35 participants. The trained SVM was then tested on the data of Experiment 2 of the left out participant. This procedure was repeated until the data of each participant was once used as test data.

The statistical analysis of the MVPA at subject-level was implemented again on the results of the MVPA performed at group-level.

## Results

### Behavioral results

During the experiment, the participants saw pictures of different faces (artificial faces in Experiment 1 and natural faces in Experiment 2). The faces expressed an ambiguous emotion and the participants had to rate these emotional expressions as positive or negative. We expected to see a negativity bias in the face ratings and this assumption was confirmed in our behavioral data, i.e. more faces were rated as negative in both experiments and this negativity bias was highly variable across participants.

**Figure 2:**
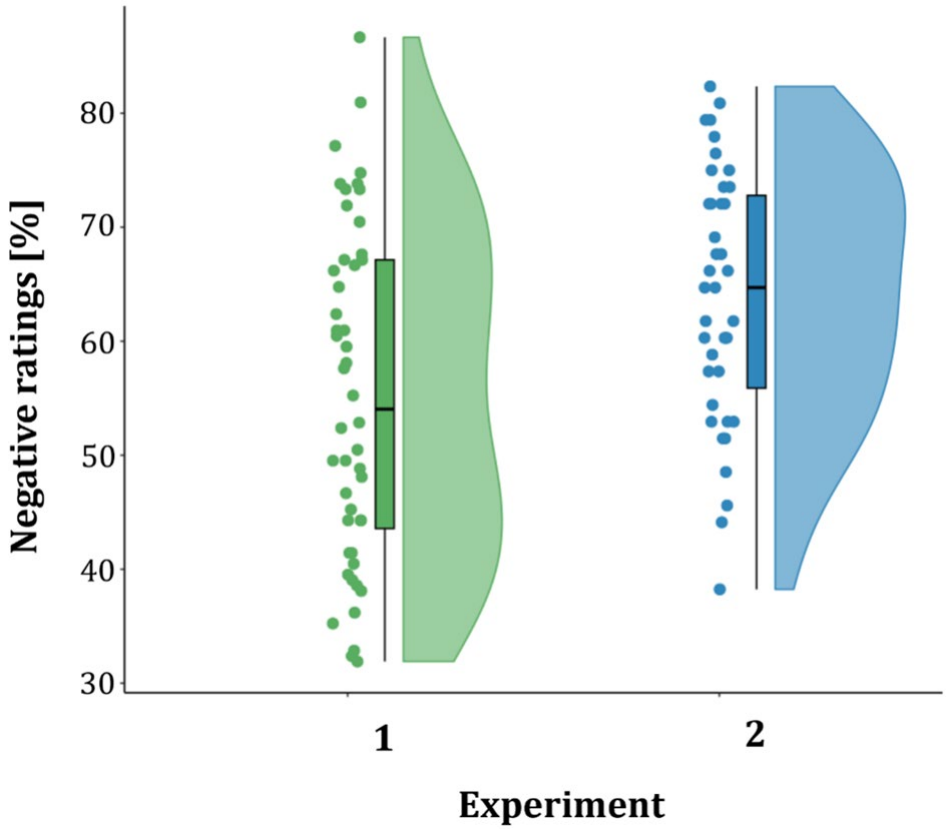
The distribution of negative ratings in percent from all ratings for Experiment 1 (N = 48; data range = 32–87%; left) and Experiment 2 (N = 39; data range = 38–82%; right) faces that expressed ambiguous emotions. Each dot represents the negative rating in percent from all ratings of a participant.

The mean percentage of negative ratings in percent from all ratings over all participants, i.e. the negativity bias, was lower for Experiment 1 (M = 55%, *SD* = 14%; data range = 32–87%; N = 48; see Fig. 2) compared to Experiment 2 (M = 65%, *SD* = 11%; data range = 38–82%; N = 39; see Fig. 2). An overall wide spread distribution across the participants’ ratings in both experiments was observed (see Fig. 2). The group statistic over both experiments showed a significant difference between the negativity bias in the two experiments (*t*_*35*_ =  − 3.77; *p* < 0.001).

Since depression as well as anxiety are known to influence emotion processing, each participant filled out both the BDI-2 as well as the STAI-Y questionnaires^23,25^. The mean scores for the questionnaires are displayed in Table 1. The potential range for the BDI-2 scores are 0 to 63, while in our sample, the scores ranged from 0 to 12. As for the STAI-S and STAI-T, their possible ranges are 20–80 respectively. In our sample, the STAI-S scores ranged from 20 to 41, and the STAI-T scores ranged from 20 to 44.

**Table 1:** Mean and standard deviation of the BDI-2 and STAI-S/STAI-T subscales.

Experiment	Questionnaire		
	BDI-2	STAI-S	STAI-T
1 (artificial)	4.04 ± 3.33	30.6 ± 5.42	30.58 ± 6.71
2 (natural)	4.13 ± 3.29	31.18 ± 5.82	31.56 ± 7.81

As the group sizes differed between the experiments, the means and standard deviations were calculated for each experiment separately. The potential range for the BDI-2 scores are l0 to 63, while in our sample, the scores ranged from 0 to 12. As for the STAI-S and STAI-T, their possible ranges are 20–80 respectively. In our sample, the STAI-S scores ranged from 20 to 41, and the STAI-T scores ranged from 20 to 44.

To test if there is an association between the negativity bias and depressive or anxious tendencies, we computed a Pearson correlation coefficient between the negativity bias and the BDI-2/STAI-S/STAI-T scores. We found no significant correlation between the ratings and the BDI-2 or STAI-S/STAI-T scores in both experiments (see Table 2).

**Table 2:** Results of the computed Pearson correlation coefficient between the BDI-2 as well as STAI-S/STAI-T subscales with the negative ratings in percent from all ratings for Experiment 1 (artificial faces) and Experiment 2 (natural faces). Displayed are the correlation coefficient values.

Experiment	Questionnaire		
	BDI-2	STAI-S	STAI-T
1 (artificial)	− 0.175	0.106	0.149
2 (natural)	0.041	− 0.21	− 0.081

### Electrophysiology results

**Figure 3:**
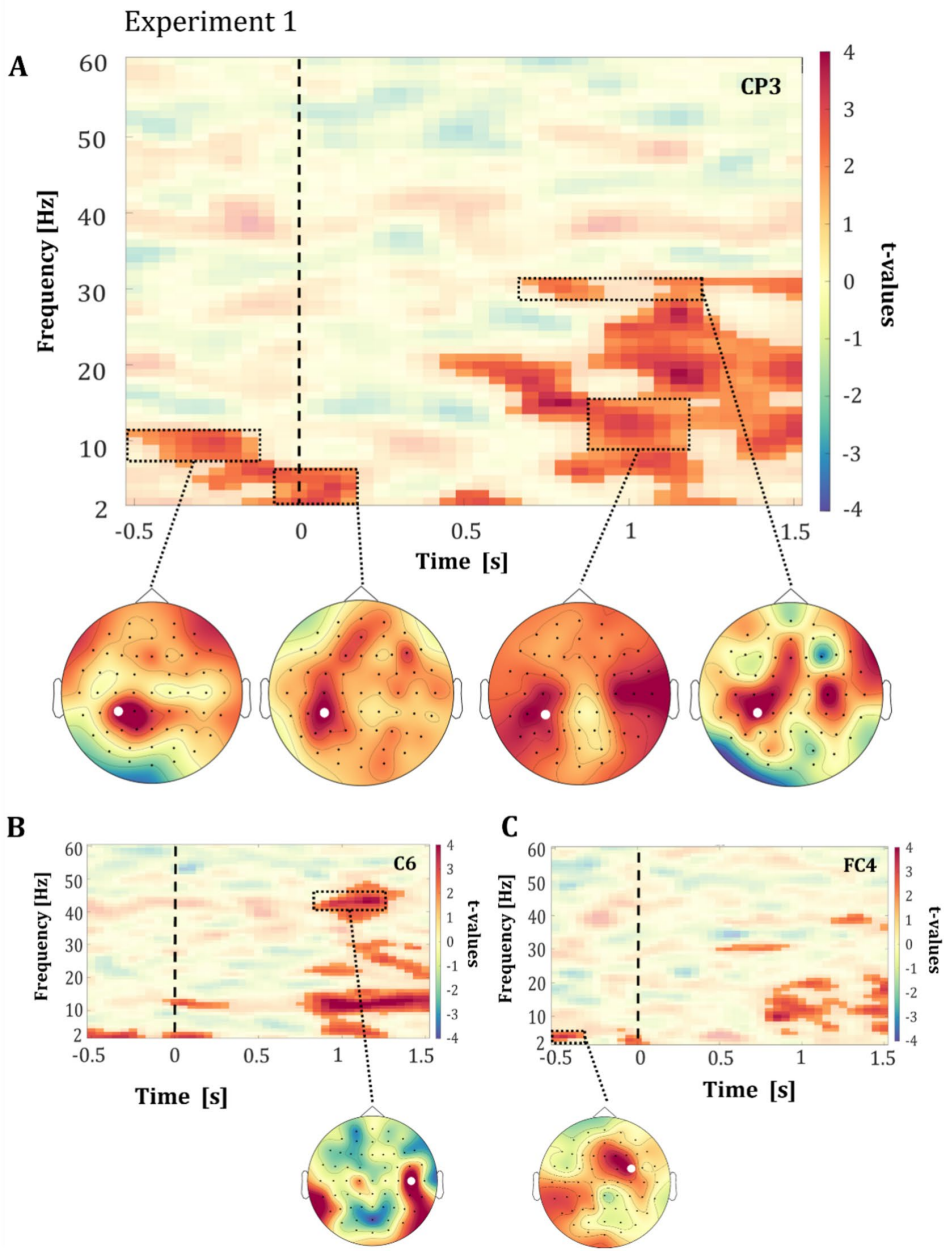
Pre- and post-stimulus results from Experiment 1. Time–frequency representations (TFRs) showing the t-values of difference in power between negatively and positively rated facial expressions across all participants in (**A**) channel CP3, (**B**) channel C6, and (**C**) channel FC4. The facial stimulus was presented for 200 ms with its onset at 0 ms, marked by a dashed line. The motor response was required from 2000 ms after stimulus onset onwards. The highlighted areas represent the significant t-values (*p* < 0.05, corrected) and non-significant t-values are transparent. The topographic plots show the spatial distribution of the t-values in the respective time–frequency windows, which are marked with a rectangle. The white dot in the topographic plots marks the position of the channel that is shown in the TFR above.

**Figure 4:**
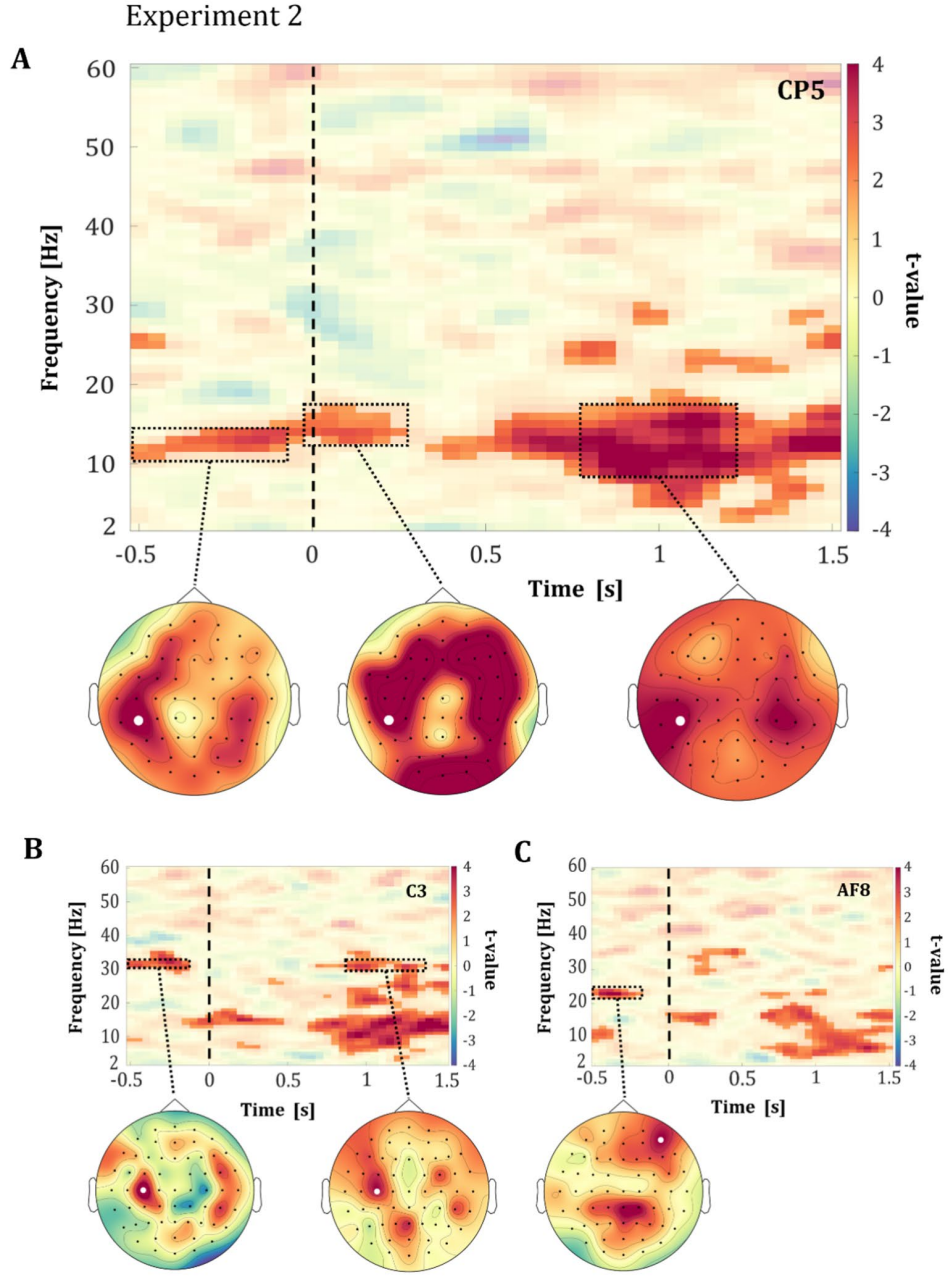
Pre- and post-stimulus results from Experiment 2. TFRs showing the t-values of the difference in power between negatively and positively rated facial expressions across all participants in (**A**) channel CP5, (**B**) channel C3 and (**C**) channel AF8. The facial stimulus was presented for 200 ms with its onset at 0 ms, marked by a dashed line. The motoric response was required from 2000 ms after stimulus onset onwards. The highlighted areas represent the significant t-values (*p* < 0.05, corrected) and non-significant t-values are transparent. The topographic plots show the spatial distribution of the t-values in the respective time–frequency windows, which are marked with a rectangle. The white dot in the topographic plots mark the position of the channel that is shown in the TFR above.

Our EEG results show clear differences between subsequently positively and negatively rated ambiguous facial expressions in pre- as well post-stimulus time intervals. These differences in oscillatory power indicated an overall stronger effect for facial expressions subsequently rated as negative. The cluster-statistic of the comparison of the time–frequency spectra of the two perceived valences, resulted in one significant positive cluster in Experiment 1 (*p* = 0.0135) as well as Experiment 2 (*p* < 0.001). In Experiment 1, the cluster comprised all 60 channels and frequency ranges from 2 to 49 Hz. The peak within the pre-stimulus time-window was observed in channel FC4 (4 Hz, *t*_47_ = 3.4; see Fig. 3C), and the peak within the post-stimulus time-window was observed in channel CPz (16 Hz, *t*_47_ = 5.7). In Experiment 2, the cluster comprised all 60 channels and frequency ranges from 2 to 39 Hz. The peak within the pre-stimulus time-window was observed in channel AF8 (23 Hz, *t*_38_ = 4.6; see Fig. 4C), and the peak within the post-stimulus time-window was observed in channel F1 (16 Hz, *t*_38_ = 6.3). An overview of the results of all channels for both Experiment 1 and 2 can be found in Fig. 1 of the supplementary material.

Crucially, in both experiments, we observed an effect in the alpha-band (~ 8–12 Hz) within − 500 to 0 ms relative to stimulus onset for facial expressions latter perceived as negative (see Figs. 3A, 4A). The topographical organization of the alpha-band effect shared similarities in both experiments, as illustrated in Figs. 3A and 4A. Within the pre-stimulus period, the effect in the alpha-band was predominantly observed in the left centro-parietal regions (see Fig. 3A, exemplary channel CP3 & Fig. 4A, exemplary channel CP5). This observation suggests that the ongoing activity affects the emotional processing of latter occurring ambiguous facial expressions.

In addition to the shared pattern observed in the pre-stimulus period, differential effect-patterns between the experiments were observed in lower and higher frequencies near the alpha-band in the pre-stimulus period. In Experiment 1, the effect in the alpha-band was accompanied by effects in the theta-band in the centro-parietal and frontal regions (3–7 Hz; see Fig. 3A, exemplary channel CP3 & Fig. 3C, exemplary channel FC4), whereas for natural faces in Experiment 2, the effects were also observed with the beta-band in the centro-parietal and frontal regions (13–17 Hz; see Fig. 4A, exemplary channel CP5).

Furthermore, the presentation of natural faces in Experiment 2 was accompanied by an increase in the beta (~ 22–24 Hz) and gamma (~ 30–35 Hz) frequency bands in the pre-stimulus period, as shown in Fig. 4B,C. The increased beta-band effect was primarily located in central and right frontal regions (see Fig. 4C, exemplary channel AF8), while the increased effect in the gamma-band was mainly observed in the left centro-parietal area (see Fig. 4B, exemplary channel C3). Both effects extended over substantial parts of the pre-stimulus interval of 500 ms (see Fig. 4B,C).

In addition to these pre-stimulus effects, also early stimulus processing revealed differences between positively and negatively rated facial expressions in both experiments. In Experiment 1 the previously described effects in the theta-band extends from the pre-stimulus into an early post-stimulus time-window (~ 3–7 Hz; see Fig. 3A). In Experiment 2, the described beta-band effect also extends from the pre-stimulus into early stimulus processing (~ 13–17 Hz; see Fig. 4A). Within both experiments the topographic organizations of these effects is focused on the central and centro-parietal regions. However, these effects also spread to the frontal and occipital regions, particularly evident in Experiment 2 (see Fig. 4A, exemplary channel CP5). These early differential effects between the latter rated valences, observed in both experiments, indicate a connection between pre-stimulus and early stimulus processing regarding the processing of ambiguous emotional facial expressions.

In both experiments, large post-stimulus effects were observed starting from 500 ms relative to stimulus onset onwards, which ranged from theta to gamma frequency bands (~ 3–30 Hz; see Fig. 3A, exemplary channel CP3; Fig. 4B, exemplary channel C3). The topographic organization of these effects share similarities in both experiments. Alpha–beta-band effects were observed predominantly in central and centro-parietal regions, extending to frontal regions. Effects in the higher beta-bands (~ 30 Hz) were observed mainly in left and right central and centro-parietal regions, extending to frontal and parietal regions. In Experiment 1, we additionally observed an increase in effect size in the gamma frequency band (~ 37–49 Hz) starting at ~ 800 ms relative to stimulus onset (see Fig. 3B, exemplary channel C6). This effect was mainly observed in the right central, centro-parietal and temporo-parietal regions.

### Representation of the negativity bias

As expected, we observed a negativity bias in both experiments with a wide spread distribution of ratings across the participants. We used this information to evaluate the neural representation of the negativity bias across both experiments. Specifically, the relationship between the percentage of total negative ratings per participant and the difference in the differential time–frequency spectrum of negatively and positively perceived facial expressions was computed. The two experiments were combined despite the different strength of negativity bias and the likely differences in the processing of the stimuli. This was done with the intention of investigating a potential general correlation between negativity bias and neural oscillations in the perceived valence of emotional facial expressions, specifically focusing on the pre-stimulus period, irrespective of the stimulus. As for all comparisons, we used an equal number of trials per participant for positive and negative ratings to avoid an influence of differences in trial numbers on the statistical analyses (see “Materials and methods” section for details). The cluster-statistic of the relation between the negative ratings and the time–frequency decomposed data of the differently perceived valences resulted in one significant positive cluster (*p* < 0.001). The cluster comprised all 60 channels and spanned the frequency range from 2 to 60 Hz. The peak in this cluster within the pre-stimulus time-window was observed in channel PO3 (57 Hz; *r* = 0.35), the peak in the cluster within the post-stimulus time-window was observed in channel P8 (11 Hz; *r* = 0.51). An overview of the results of all channels can be found in Fig. 1 (lower plot) of the supplementary material.

We observed clear positive correlations between the negativity bias and the oscillatory power in broad time-intervals before and after stimulus presentation over theta to gamma frequency bands.

**Figure 5:**
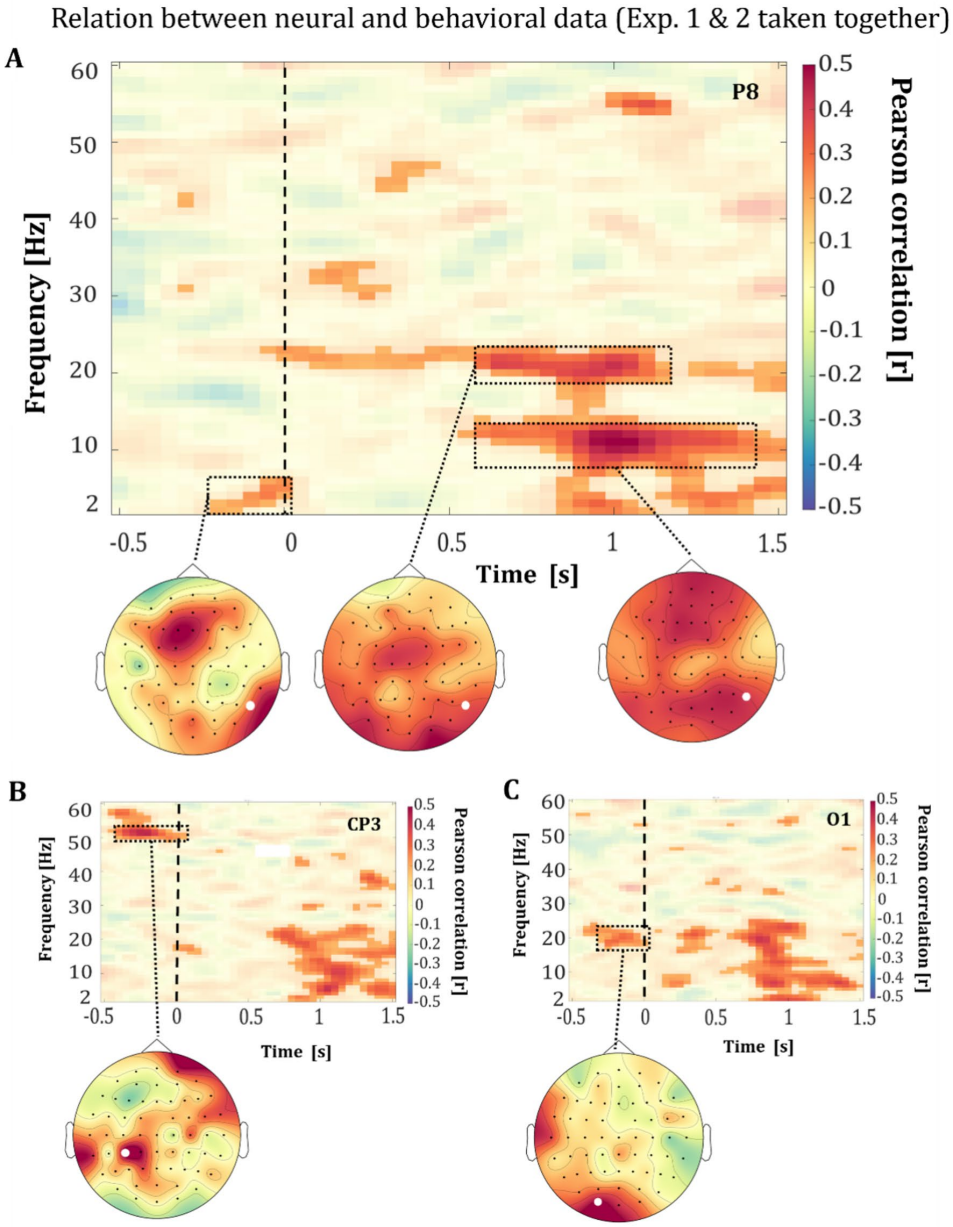
Relation between the neural and behavioral data over both experiments. TFRs demonstrating the representation between the negative ratings of facial expressions in percent in relation to all ratings and the difference (negatively and positively rated facial expressions) in oscillatory power of the face ratings in (**A**) in channel P8, (**B**) channel CP3, and (**C**) channel O1. The facial stimulus was presented for 200 ms with its onset at 0 ms**,** marked by a dashed line. The motor response was required from 2000 ms after stimulus onset onwards. The highlighted areas represent the significant correlation coefficients (*p* < 0.05*,* corrected) and non-significant correlation coefficients are transparent. The topographic plots show the spatial distribution of the correlation coefficients in the respective time–frequency windows, which are marked with a rectangle. The white dot in the topography marks the position of the channel that is shown in the TFR above.

Within the pre-stimulus period a relation of the negativity bias with the oscillatory activity was observed in the theta-band (~ 3–7 Hz) within parietal and frontal regions (see Fig. 5A, exemplary channel P8), in the beta-band (~ 17–23 Hz) within occipital and temporal regions (see Fig. 5C, exemplary channel O1) and higher gamma-band (~ 50–60 Hz) within centro-parietal, temporo-parietal and frontal regions (see Fig. 5B, exemplary channel CP3). These effects were observed in the 500 ms preceding stimulus onset (see Fig. 5A–C).

In addition to the evident pre-stimulus effects, we also observed a positive association of neural activity with negativity bias within the post-stimulus interval. We observed broad effects starting at ~ 600 ms relative to stimulus onset within the alpha- (8–13 Hz) and beta-band (19–24 Hz) (see Fig. 5A). The post-stimulus patterns illustrating the relationship between the negativity-bias and the oscillatory difference in perceived valence share similarities with the post-stimulus effects observed in the time–frequency decomposed data of the oscillatory differences in perceived valence (see Figs. 3A, 4B). This strongly supports the assumption that processing of emotional faces may be modulated by a negativity bias.

For the alpha-band effect, we observed a broad topographic distribution with prominent extension over the parietal, centro-parietal and frontal regions (see Fig. 5A, exemplary channel P8). For beta-band effects, we observed a broad topographical distribution with prominent extension over the frontal, fronto-central, parietal, and occipital regions (see Fig. 5A, exemplary channel P8).

### MVPA results

To evaluate the relevance of pre-stimulus oscillatory activity for the subsequent processing of emotional expressions of differently perceived valences, two MVPA approaches were applied, one at the subject and one at the group-level. Both MVPA analyses utilized a cross-decoding approach.

**Figure 6:**
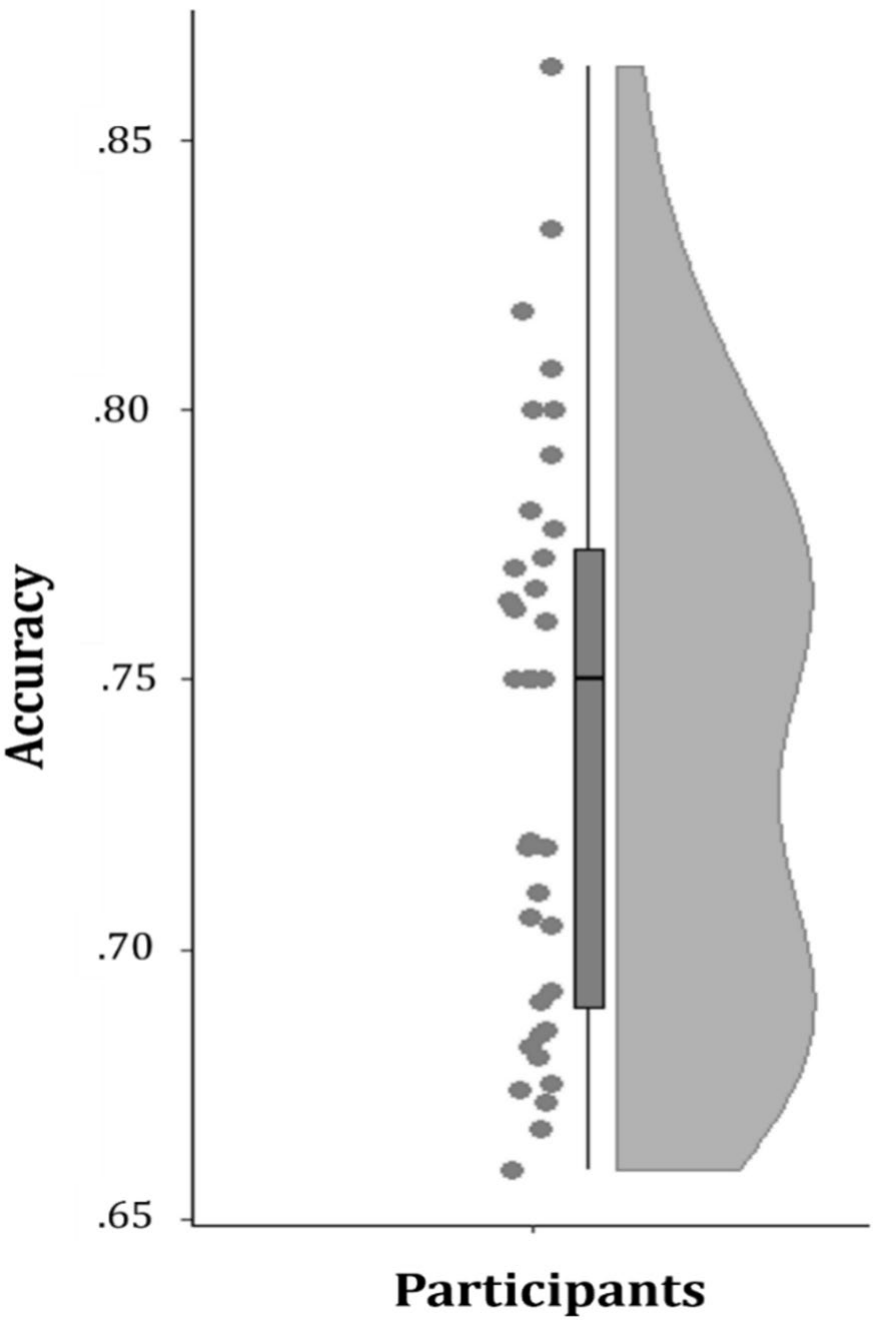
The distribution of peak accuracies per participant in the binomial statistic obtained from the within subjects cross experiment decoding approach. Each dot represents the peak accuracy of a participant (data range = 0.66–0.86). The results indicate that cross-decoding performed well, with accuracies above chance level (0.5) for each participant and across participants (*t*_35_ = 26.856, *p* < 0.001).

In the MVPA at the subject level, a SVM was trained on the time–frequency decomposed data of each participant separately in Experiment 1. Subsequently, we tested this trained SVM on the time–frequency decomposed data for each participant separately in Experiment 2. This analysis aimed to ascertain whether similar patterns existed within participants and across both experiments, i.e. irrespective of the implemented stimuli, during the pre-stimulus time-window (− 1000 to − 200 ms) without any overlap of the post-stimulus window. The results of our analysis indicated that the SVM trained on the time–frequency patterns preceding the subsequently negatively and positively rated faces from Experiment 1 was able to accurately decode the patterns preceding the subsequently negatively and positively rated faces from Experiment 2. The peak accuracy was above a chance level of 0.5 for each participant (see Fig. 6). The mean peak accuracy across the entire group (N = 36) was 0.739 (*SD* = 0.053; data range = 0.66–0.86). Furthermore, the peak accuracies across all participants were found to be significantly different from chance level (*t*_35_ = 26.856, *p* < 0.001).

To exclude a possible contamination of the pre-stimulus period by oscillatory activity related to the perceived valence of the previous trial, we performed an MVPA. We used a cross-decoding approach to the data from Experiment 1 (N = 36), which had already been used in the MVPAs performed at the subject and group level.

First, trials with neutral or missed previous ratings were excluded from analyses. Then we used the labels of the rating from the previous trial to train an SVM (metric = accuracy). We then tested the trained SVM using the current rating labels of the trials. This approach allowed us to investigate whether an SVM trained on oscillatory data labeled with the previous trial ratings could successfully decode the pre-stimulus data with the correct labels, thereby assessing the influence of the previous rating on the current rating.

To evaluate the performance of this MVPA, we conducted a second MVPA using the labels of the current ratings for both training and testing an SVM (metric = accuracy; cross-validation = k-folds with 5 folds and 4 repetitions).

Mean peak accuracy across the entire group in the MVPA trained on the labels of the previous ratings performed within participants of Experiment 1 was 0.547 (chance level = 0.5, SD = 0.048). Mean peak accuracy across the entire group in the MVPA trained on the labels of the current ratings performed within participants of Experiment 1 was 0.650 (SD = 0.045). The results of the paired t-test conducted between these MVPAs were found to be statistically significant (t_35_ = 8.131, p < 0.001). We conclude that the activity measured in the pre-stimulus period is not significantly influenced by the previous stimulus evaluation. This conclusion is supported by the worse performance of the MVPA trained with the labels of the previous ratings compared to the MVPA trained with the labels of the current ratings, as well as the significant difference in performance between these two MVPAs.

In the MVPA performed at the group level, a cross-decoding approach utilizing LOPOCV was applied on exclusively pre-stimulus oscillatory activity. This analysis aimed to ascertain whether similar patterns existed across participants and experiments. The same time interval (− 1000 to − 200 ms) utilized in the MVPA at subject-level was employed. Using a cross-decoding approach, we trained a SVM on the time–frequency decomposed data using a LOPOCV approach. This means, that the SVM was trained on the pooled data of Experiment 1 of 35 participants. The trained SVM was then tested on the data set of the remaining participant with data of Experiment 2. This was repeated until the data of each participant was used as test data set. The peak accuracy was above chance level of 0.5 for each iteration. The mean peak accuracy across all iterations was 0.69 (*SD* = 0.056; data range = 0.58–0.82). Furthermore, the peak accuracies across all iterations were found to be significantly different from chance level (*t*_35_ = 21.4, *p* < 0.001) again indicating a general relevance of pre-stimulus activity for the perceived valence of different face stimuli.

## Discussion

The objective of our study was to examine the relevance of pre-stimulus oscillatory activity for the processing of latter presented emotional facial expressions of differently perceived valences. The results showed differences between subsequently perceived emotional valences in the oscillations before the presentation of the facial expression, as well as early and late effects following stimulus onset.

The involvement of pre-stimulus activity in the processing of emotional faces was replicated using different stimulus material**.** The relevance of this activity was demonstrated through two multivariate approaches applied selectively on the pre-stimulus data, with one experiment serving as the training dataset and the other as a test dataset.

Finally, across the participants the observed behavioral negativity bias was also positively related to neural oscillations in the pre-stimulus period, i.e. prior to the appearance of the stimulus, underscoring the significance of ongoing activity for latter emotional processing.

### Perceived valence of latter presented emotional expressions is influenced by pre-stimulus oscillations

As hypothesized, we observed late and early post-stimulus effects triggered by emotional facial expressions of latter rated positive and negative valence in both experiments. Such subsequent stimulus effects were also reported by previous EEG studies on the processing of emotional facial expressions^5,11,12,14,18–20^. The disparities in oscillations, which we observed between negative and positive emotional facial expressions, particularly in the later post-stimulus time window within the alpha to beta bands in both experiments, could be attributed to differences in processing the perceived valences. Previous research using images from the International Affective Picture System (IAPS), has shown that stimuli with a more negative valence tend to produce stronger effects in the alpha and beta bands^63,64^**.**

Importantly, our study adds a novel finding: We observed valence-dependent differences in pre-stimulus activity for subsequently presented ambiguous facial expressions. These differences in pre-stimulus activity were observed for both artificial facial expressions in Experiment 1 and natural facial expressions in Experiment 2. Our finding indicates the potential relevance of pre-stimulus neural dynamics in modulating subsequent emotional processing. The modulatory role of oscillations on a subsequent stimulus has been already proven in various studies^31,33–36,65,66^. For instance, in the field of memory, it has been discovered that pre-stimulus oscillations play a role in influencing the successful encoding of subsequent stimuli^34,66,67^. The causal role of pre-stimulus oscillations on subsequent stimulus modulations was further supported by studies successfully using a brain computer interface to enhance memory encoding and visual perception of subsequent stimuli^34,35^.

Our study extends this understanding to the domain of emotional processing, demonstrating the influence of pre-stimulus oscillations on the processing of emotional facial expressions. Notably, our results emphasize the role of the alpha band in processing emotional facial expressions of differently perceived valences. We observed significant valence-dependent differences in oscillatory activity preceding the visual stimulus in the alpha band (~ 8–12 Hz) across both experiments. The alpha band was initially believed to primarily play a role in simple visual processing, with alpha synchronization occurring when the eyes are closed and alpha de-synchronization, i.e. suppression, in response to opening the eyes^68^. However, research over the past decades has shown that alpha band activity is involved in several cortical processes such as long-term memory^69^, or modulation of visual perception^33,70^. Furthermore, previous research on affective stimuli^64,71^ and face^30^ processing has elucidated the involvement of alpha oscillations in predictive processing. In our study, however, there was no experimental manipulation of the expectation of the valence of facial expressions. Although not definitive, it can be speculated that the differentiation observed in the alpha band prior to the stimulus was caused by individual, valence-dependent fluctuating expectations. On the other hand, the effects observed during the pre-stimulus period, could also originate from fluctuations in emotional states. This theory is supported by a study that examined spontaneous emotional states during resting-state. Kragel et al. conducted an fMRI study in which they successfully decoded distinct emotional states occurring spontaneously during resting-state^72^. We therefore assume that spontaneous fluctuations in emotional states could influence subsequent processing of facial expressions in our study and are related to the effects observed in the oscillatory pre-stimulus activity.

Effects within regions previously identified in studies focusing on post-stimulus emotion processing, including frontal, central, centro-parietal, temporal and occipital regions^8,9,19^ were observed in our study in pre- as well as post-stimulus periods. Despite numerous studies on processing emotional facial expressions, it remains speculative whether the effects we observed during the pre-stimulus period originate from the same regions observed in direct stimulus processing. Considering that participants were instructed to provide a motor response 2000 ms after the visual stimulus onset, and trials involving a motor responses prior to 200 ms before the requested response were excluded, it is reasonably to concluded that the observed results are not attributable to motor responses.

In addition to the commonalities observed across experiments, we also found differences in activity during the pre-stimulus interval between experiments. In Experiment 1 alpha band effects accompanied by theta oscillations were observed, while Experiment 2 showed alpha effects accompanied by beta and gamma oscillations. It is unlikely that these differences were caused by differences of the processed emotions, as both stimulus sets comprised surprised faces. The disparities observed in the pre-stimulus oscillations across our experiments could potentially stem from the contrast between the use of artificial faces as stimuli in Experiment 1 and natural faces in Experiment 2. This assumption is supported by findings of an EEG study in which less arousal for artificial faces was observed ^73^ and an fMRI study in which less activity in the amygdala for artificial faces compared to natural human faces was observed ^74^. Furthermore, in Experiment 1, the same 14 faces were presented throughout, while in Experiment 2, each face image was distinct, which could have established differential cognitive settings that may affect pre-stimulus effects. Nevertheless, it is noteworthy that common effects emerged across both experiments during both the pre-stimulus and post-stimulus phases.

To assess both the similarities and differences between the two experiments and to evaluate their statistical relationship, as well as to gage the generalizability of our pre-stimulus effects, we employed two different multivariate approaches. One MVPA was used to test for experiment-independent differences at the subject-level, while a second MVPA was implemented to test for experiment and subject independent differences at the group level. Specifically, this latter approach tested whether there is an overarching pattern within the pre-stimulus window that is consistent across both experiments and subjects, in relation to the valences of the subsequently rated emotional facial expressions. In both approaches we utilized data from one experiment for training and data from the other experiment for testing purposes. With both MVPA approaches accuracies significantly differing from chance level were observed.

The multivariate approach takes advantage of unique individual differences that a univariate analysis cannot identify. Emotion processing is subject to inter-individual differences, as shown by studies on mood-related disorders ^25,75,76^ and healthy participants ^26,40^. In addition, a variety of different processes are involved in the processing of emotions ^77,78^. However, both MVPAs, at subject- and at group-level, showed accuracies significantly differing from chance level. Remarkably, we observed reliable classification above chance for all participants over a pre-stimulus time window of 1000 ms, with a mean accuracy of 0.739 within subjects and 0.699 on the group-level. With the use of multivariate approaches we were able to highlight the generalizability of our results across different stimulus sets as well as participants, providing further evidence for the modulatory role of pre-stimulus oscillations on processing of emotional facial expressions. Our findings allow us to assume that overarching patterns exist in oscillations during the pre-stimulus period between differently perceived valences of emotional facial expressions.

### Representation of the negativity bias

As hypothesized, we observed a negativity bias in valence ratings across both natural facial expressions in Experiment 1 and artificial facial expressions in Experiment 2. The observed bias in our study is comparable with findings of other studies on surprised faces. We found a mean of 55% and 65% for negative ratings of artificial faces in Experiment 1 and natural faces in Experiment 2 respectively. In their 2018 study on individual differences in the valence bias, Petro and colleagues reported a valence bias of natural surprised faces with a mean of 59.1% negative ratings in 57 participants ^40^. In their 2021 study on individual differences in response to emotional ambiguity, Neta and Brock reported 63.59% negative ratings for surprised faces in natural faces ^26^. Therefore, we see a typical strength of the negativity bias in healthy participants for surprised facial expressions.

The neural data reflected the observed negativity bias, evident in the positive association of negative valence ratings with the effect in neural activity between the valence ratings. This positive association spanned over both early and late post-stimulus time frames and across all frequency bands from theta to gamma (see Fig. 5A). This finding suggests that the processing of emotional facial expressions is modulated by the negative bias. Supporting this interpretation, an fMRI study revealed a correlation of the negative bias with the neural activity in the left middle frontal cortex, which exhibited a stronger effect with higher overall negative ratings of surprised faces compared to neutral faces ^40^. Moreover, we found a positive relationship between the negativity bias and pre-stimulus oscillations, suggesting that stimulus-independent oscillations already initiated biased processing of perceived valence (see Fig. 5A–C). The concept of the negativity bias has spurred various theoretical frameworks ^40,79,80^. One prominent theory, the initial negativity hypothesis, posits that emotional facial expressions with an inherent ambiguity like surprise are initially processed as negative, with subsequent cognitive processes potentially shifting valence judgments towards positivity ^40^. We propose that stimulus-independent oscillations observed in the pre-stimulus period may further modulate the process described in the initial negativity theory, which suggests that ambiguous emotional expressions are automatically processed as negative, followed by a cognitive switch for positively interpreted ambiguous faces ^5,11,40,41^.

Our study uncovered differences in valence perception related to negative bias during the pre-stimulus phase among healthy participants, alongside individual variations in biased behavior favoring negativity. This prompted the question of what factors may contribute to such biases in our data. The strong influence of bias in affective disorders, in which a high sensitivity for sadness was observed, highlights the impact of mood on biased interpretations of facial expressions ^25,43^. Whereas the efficacy of mood interventions on negative biases in both clinical ^42,45^ and healthy populations ^27^ underscores the dynamic nature of affective processing. Interestingly, our findings indicate that the observed positive association of neural activity with negativity bias was mood independent, as evidenced by the non-significant associations of the negativity bias with the STAI-S/-T and BDI-2 scores. This suggests that the effects observed in our study may arise from trial-by-trial fluctuations in oscillatory states, directly impacting biased processing of subsequent ambiguous facial expressions. While mood undoubtedly contributes to bias modulation, our findings suggest that fluctuations between probably affective states may also exert a substantial influence on shaping interpretation biases. However, it remains unclear which factors, beyond mood, may influence these fluctuations. In the context of interpretation bias concerning ambiguous facial expressions in healthy participants, recent studies have shed light on the significant influence of various factors, including social connectedness, stress and emotional regulation abilities on the interpretation bias ^26,27^. For instance, it is hypothesized that heightened regulatory capabilities may result in a tendency for more positively biased interpretations of ambiguous facial expressions, while individuals with lower regulatory skills may exhibit the opposite inclination ^26,27,40^. Moreover, these regulatory abilities are likely correlated with a higher overall capacity to cope with stress, whereby the latter has also been associated with a greater negativity bias ^27^. Even though both anxiety as well as depression tendencies did not correlate with the negativity bias in our study, stress and the ability to regulate ones emotions might have been a contributing factor to the neural effects of biased valence ratings observed in this study.

Further research should examine how pre-stimulus activity influences the subsequent processing of emotional stimuli, with a particular focus on frequencies and how they influence the effect. And also how pre-stimulus activity interacts with the interpretation bias should be studied in order to comprehensively explore the underlying mechanisms.

## Conclusion

The processing of emotions is inherently complex. In our study, we identified both post- as well as pre-stimulus variations of neural oscillations between positively and negatively perceived valences of emotional facial expressions. We reliably replicated these findings using two separate sets of stimuli featuring surprised facial expressions. Our study demonstrates that in addition to stimulus-driven mechanisms, neural activity preceding the stimulus likely contributes to how the valence of subsequent emotional facial expressions is processed. This relationship likely influences early post-stimulus processes, but possibly also late post-stimulus processes. This is supported by our replication of the results using two separate stimulus sets in two experiments, one featuring artificial expressions and the other natural expressions. Furthermore, our successful decoding analyses on both experiments at subject- as well as at group-level indicates that the observed pre-stimulus oscillations are associated with perceived emotional valence. Additionally, our findings suggest that pre-stimulus neural dynamics are associated with a biased perception of emotional valence, indicating that biases may arise before stimulus onset and influence subsequent emotion processing. Our observations highlight that pre-stimulus oscillations are relevant for the processing of subsequent emotional facial expressions and that there is a necessity of further investigations on the role of preceding oscillations on the processing of emotions.

### Supplementary Information

Supplementary Information.

## Data Availability

The datasets generated and/or analyzed during this study are available on request from the corresponding author.

## Abbreviations

BDI-2: Beck depression inventory 2
dB: Decibel
EEG: Electroencephalography
fMRI: Functional magnetic resonance imaging
ITI: Inter trial interval
LOPOCV: Leave-one-participant-out cross-validation
LPP: Late positive potential
MVPA: Multivariate pattern analysis
SD: Standard deviation
STAI: State trait anxiety inventory
TFA: Time frequency analysis
