# Supplementary material for: Relevance of pre-stimulus oscillatory activity for the perceived valence of emotional facial expressions
Source: Sci Rep. 2024 Aug 20;14:19263. doi: 10.1038/s41598-024-69433-0 (PMC11336227; doi:10.1038/s41598-024-69433-0)
Supplement: Supplementary file 1 — Supplementary Information. [file 41598_2024_69433_MOESM1_ESM.docx]

**Supplemental Information**

Experiment 1

**
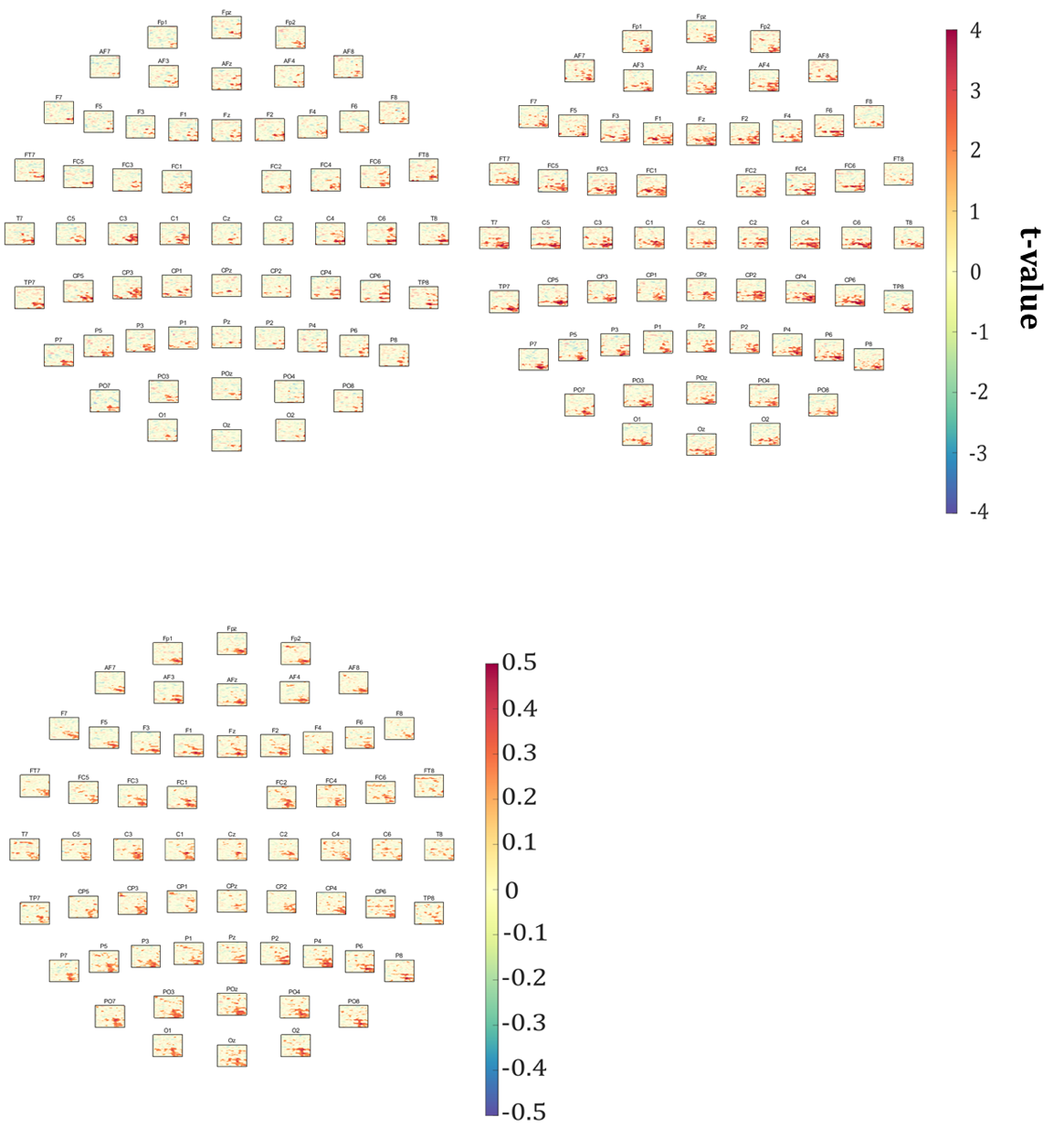

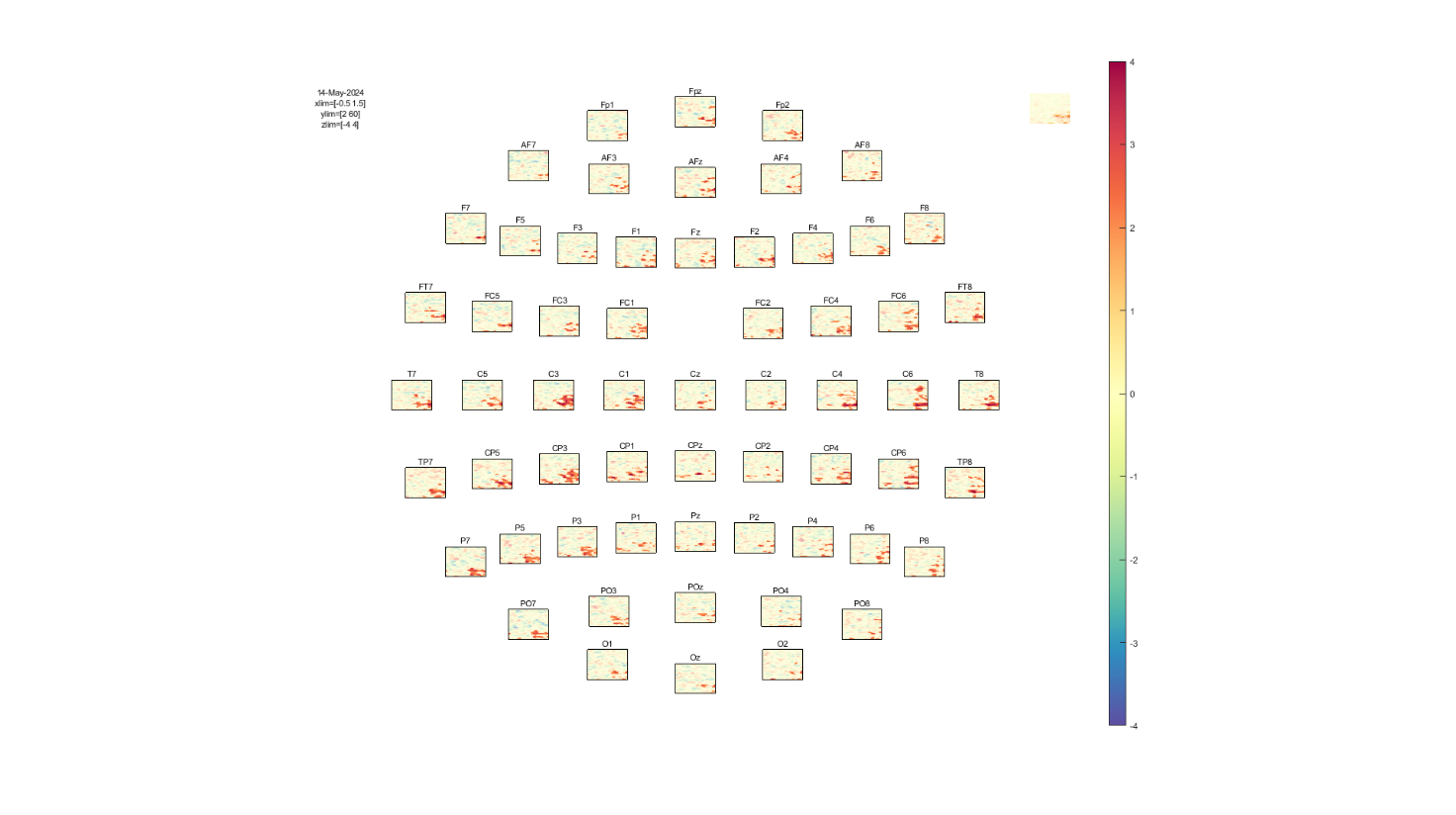
**

Experiment 2

**
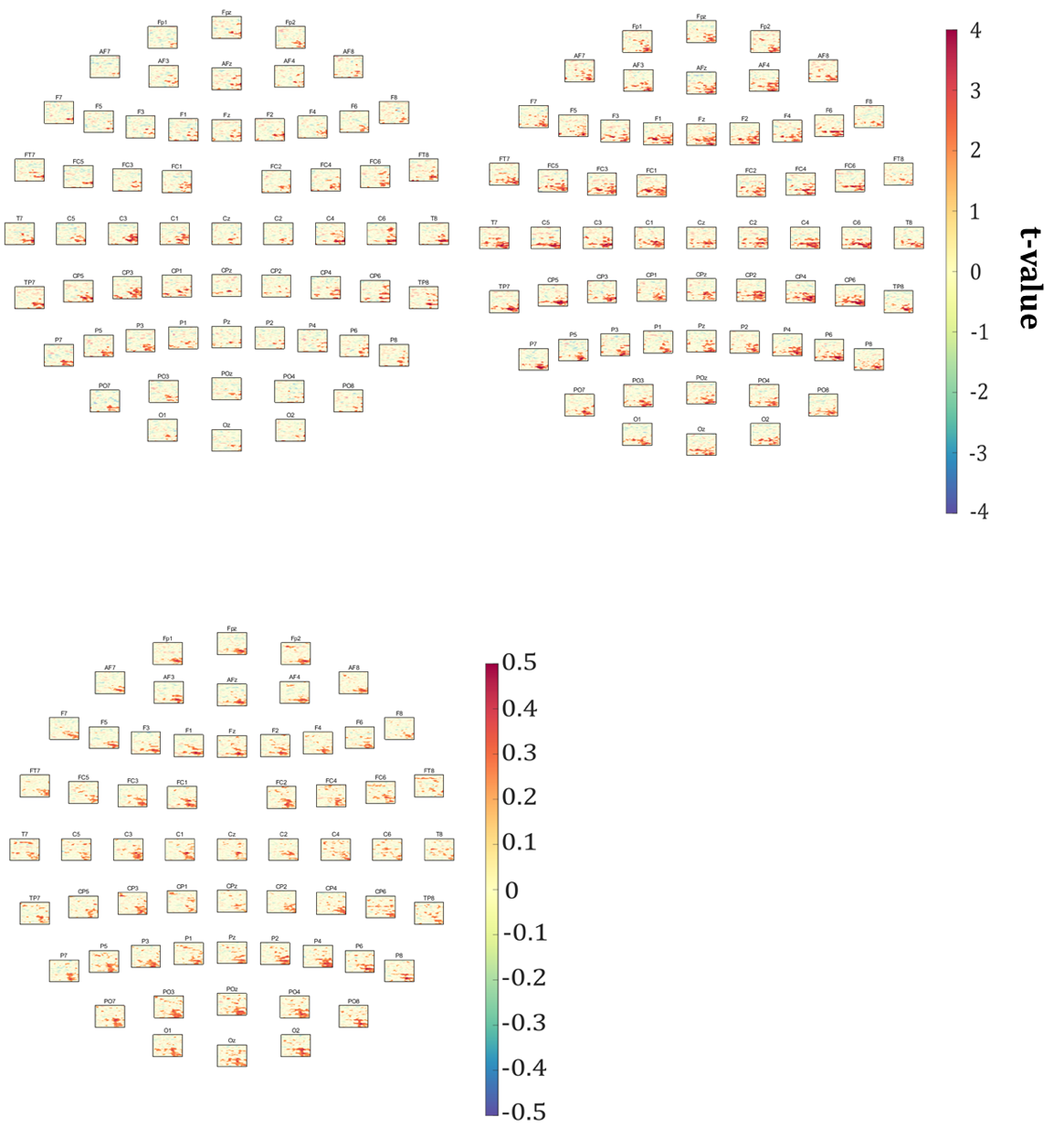
**
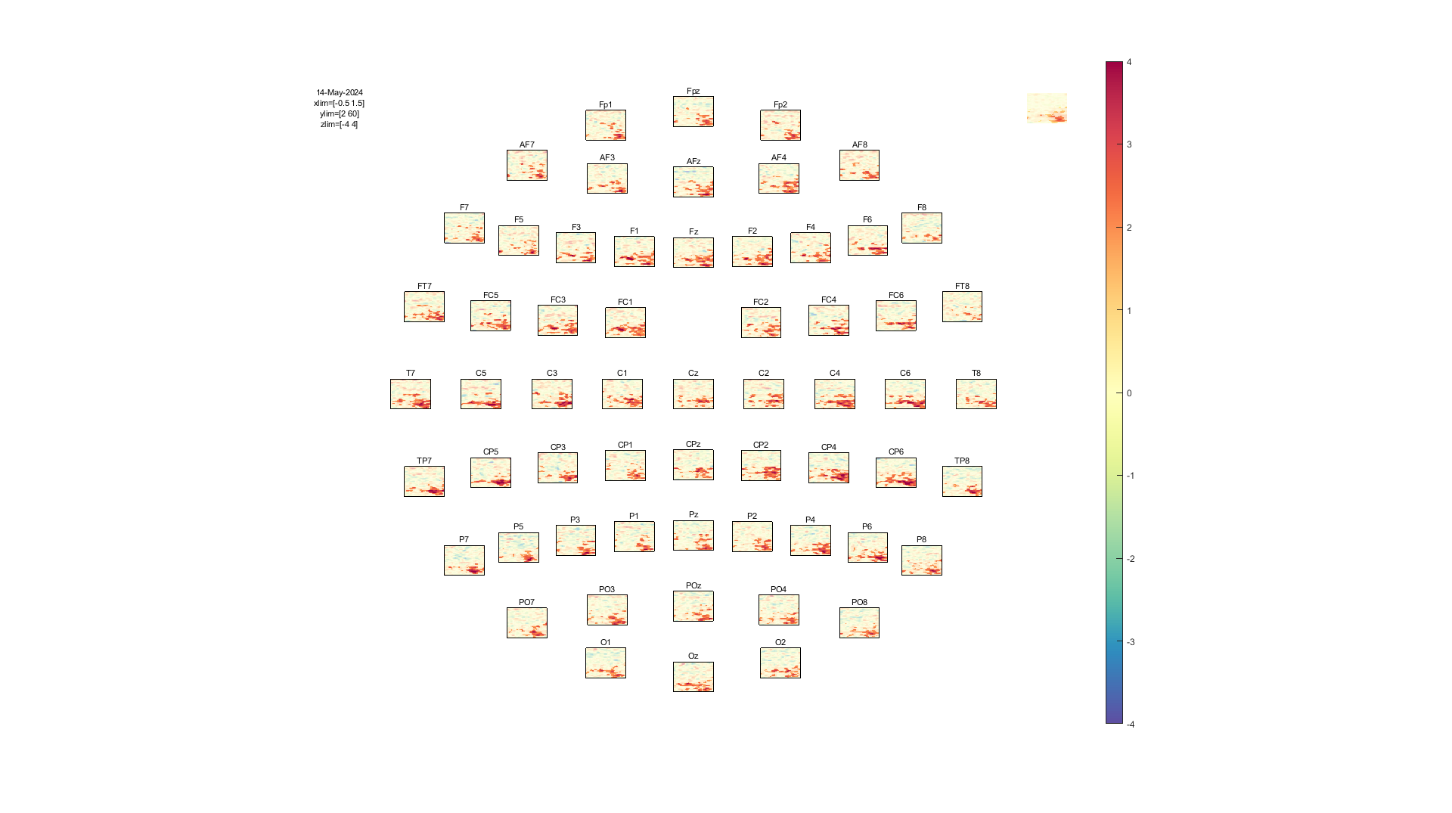


**
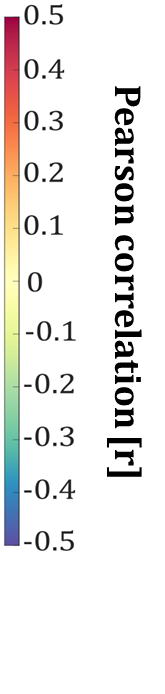
**
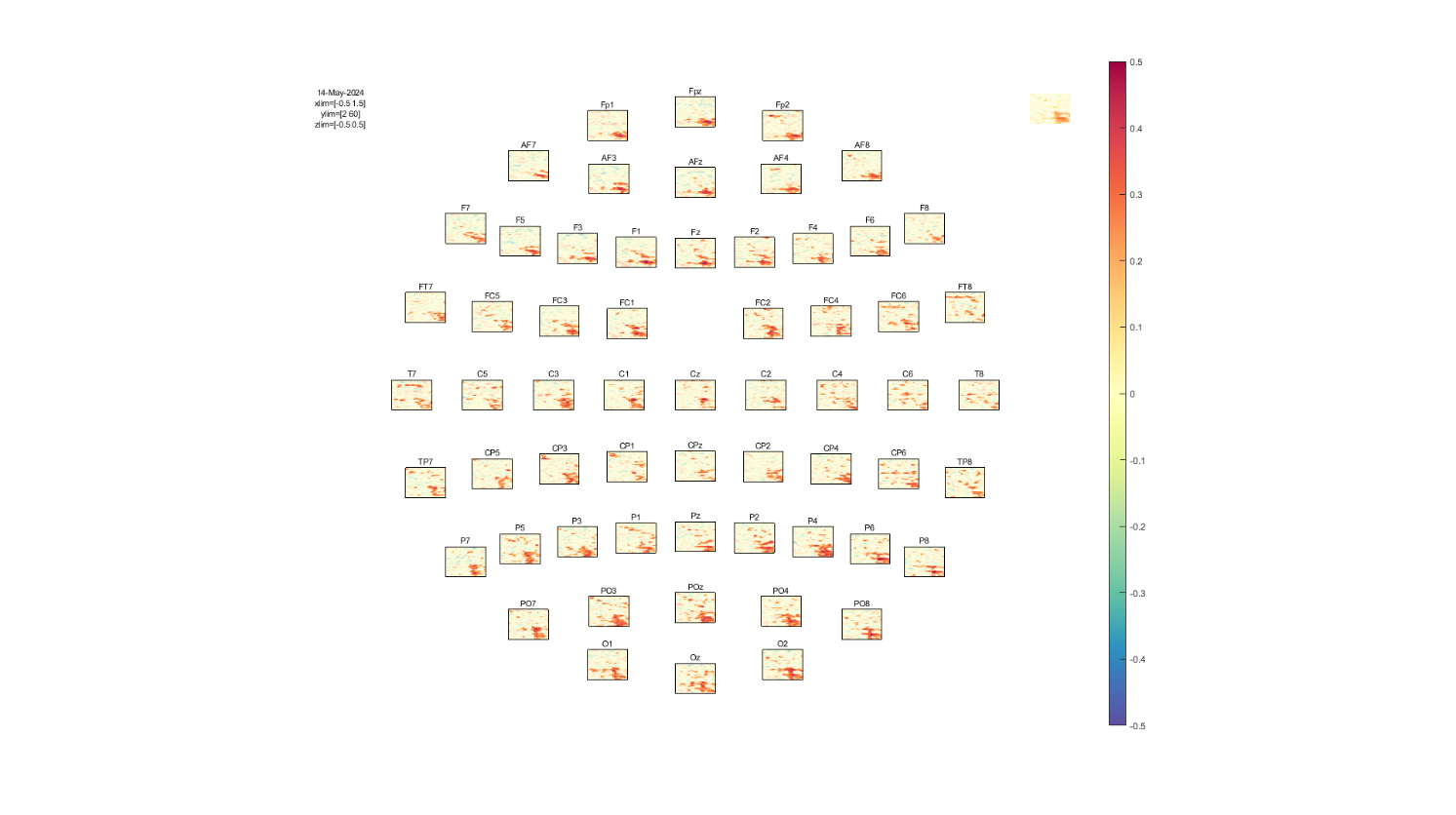


Relation between neural and behavioral data (Exp. 1 & 2 taken together)

**Supplementary Figure 1: Multiplots resembling the results of all channels. Pre- and post-stimulus results from Experiment 1 (upper) & 2 (middle) as well as the relation between neural and behavioral data (lower) are presented. The TFRs in the upper and middle multiplot show the difference between negatively and positively rated facial expressions across all participants. The TFRs in the lower multiplot show the association between the negative ratings of facial expressions in percent in relation to all ratings and the difference (negatively and positively rated facial expressions) in oscillatory power of the face ratings (negative – positive power spectrum). The highlighted areas in the upper and middle TFRs represent significant t-values (*p* < .05, corrected t-values; non-significant t-values are transparent). The highlighted areas in the lower TFRs represent significant correlation coefficients (*p* < .05*,* corrected; non-significant correlation coefficients are transparent).**
